# Individualized feedback during simulated laparoscopic training: a mixed methods study

**DOI:** 10.5116/ijme.55a2.218b

**Published:** 2015-07-29

**Authors:** Liv Ahlborg, Maria Weurlander, Leif Hedman, Henry Nisell, Pelle G. Lindqvist, Li Felländer-Tsai, Lars Enochsson

**Affiliations:** 1 Department of Clinical Science, Intervention and Technology (CLINTEC), Karolinska Institutet at Karolinska University Hospital, Sweden; 2 School of Education and Communication in Engineering Science (ECE), KTH Royal Institute of Technologyg, Sweden

**Keywords:** Feedback, laparoscopic simulator training, self-efficacy, flow

## Abstract

**Objectives:**

This study aimed to explore the value of indi-vidualized feedback on performance, flow and self-efficacy during simulated laparoscopy. Furthermore, we wished to explore attitudes towards feedback and simulator training among medical students.

**Methods:**

Sixteen medical students were included in the study and randomized to laparoscopic simulator training with or without feedback. A teacher provided individualized feedback continuously throughout the procedures to the target group. Validated questionnaires and scales were used to evaluate self-efficacy and flow. The Mann-Whitney U test was used to evaluate differences between groups regarding laparoscopic performance (instrument path length), self-efficacy and flow. Qualitative data was collected by group interviews and interpreted using inductive thematic analyses.

**Results:**

Sixteen students completed the simulator training and questionnaires. Instrument path length was shorter in the feedback group (median 3.9 m; IQR: 3.3-4.9) as com-pared to the control group (median 5.9 m; IQR: 5.0-8.1), p<0.05. Self-efficacy improved in both groups. Eleven students participated in the focus interviews. Participants in the control group expressed that they had fun, whereas participants in the feedback group were more concentrated on the task and also more anxious. Both groups had high ambitions to succeed and also expressed the importance of getting feedback. The authenticity of the training scenario was important for the learning process.

**Conclusions:**

This study highlights the importance of individualized feedback during simulated laparoscopy training. The next step is to further optimize feedback and to transfer standardized and individualized feedback from the simulated setting to the operating room.

## Introduction

Learning is a complex process and is of particular importance in medical disciplines, such as surgery, since it also concerns patient safety. Finding a balance between doctors' training and patients' safety demands a structured, and preferably evidence-based, approach to surgical education. Such an approach needs, among other things, to include; training of technical skills, continuous assessment of skills and also attention to non-technical skills.^1^ The learning environment can facilitate or obstruct the learning process by influencing these factors.^2^

Education of surgeons is rooted in apprenticeship methods developed over a century ago, as originally championed by William Halsted in 1904.^3^ The Halsted "*see one, do one, teach one"* surgical training method is, however, associated with unacceptable cost from a patient safety perspective. Although this approach has fostered excellent surgeons, we can suspect that some of this excellence came at the expense of the patients. There is now ample evidence to suggest that more structured training programs can, in fact, enhance patient safety.^4^^-^^6^ The teacher, not the least, may have a profound influence on the learning process.^2^ Teachers' skills, assessment of skills and feedback are factors that influence the learning process and therefore the education.^1^^,^^7^

In gynecology and general surgery, the laparoscopic approach is nowadays the first choice in the majority of, at least benign, abdominal procedures. This development has been driven by the lesser surgical trauma inflicted on the patients, faster post-operative recovery, shorter absence from work and also the fact that it is cosmetically more pleasing than traditional, open, surgery.^8^ In laparoscopy, the learning process for novices is, however, longer than in open surgery.^9^ Particular abilities are needed in laparoscopy. For example, the ability to convert the two-dimensional video-image to a three-dimensional mental picture.^10^ Therefore the curriculum in laparoscopic education demands a different set of tools compared to that of traditional open surgery. Simulators are important tools for improving the technical skills needed in laparoscopic surgery.^11^^-^^13^ A recent review by Thomas^14^, concludes that surgical simulation should be tailored to the individual needs and include feedback by tutors. Feedback has been defined as: "*Specific information about the comparison between a trainees´ observed performance and a standard, given with the intent to improve the trainees´ performance*".^15^ Feedback ought to be informative and non-judgmental. As an integral part of the learning process, such feedback allows the student to remain on course in reaching a goal.^16^ Feedback can be both positive and negative, but needs to be constructive. Feedback may, however, result in decreased self-efficacy, negative self-reactions, and decreased interest in performing the task at hand.^17^

While teacher feedback has a long-standing tradition in surgical training, it has in most settings been unstructured and informal.^18^. Inasmuch as it has been evaluated, some studies suggest that instructor feedback improves the laparoscopic result.^19^^-^^22^ Others, however, report that an independent approach, without feedback, in simulated laparoscopy, might actually better facilitate learning.^23^ Moreover, one study suggests different perceptions of feedback among teachers and students. For example, whereas 86% of surgeons felt that feedback was given often/always immediately after the activity, only 12.5% of residents agreed.^24^ Previous studies of feedback in laparoscopic simulator training, suggest that feedback is best given according to the individuals' needs.^7^^,^^25^. However, little is known on how individual feedback is best structured.

Several non-technical factors, like self-efficacy, also appear to contribute to learning in laparoscopy and simulated laparoscopy. Self-efficacy; one´s belief in one´s ability to succeed in specific situations,^26^ has been reported to be relevant for learning and to correlate with simulated laparoscopic performance among surgical residents.^27^ According to Banduras´ social cognitive theory; successful performance enhances, whereas repeated failures reduce, perceived self-efficacy.^28^ Efficacy beliefs are essential in the development of motor skills and how well they are executed under pressure. Belief in one´s abilities to learn the patterns of the action and successfully deliver them, contribute independently to better performance.^28^ The learning environment can also contribute to perceived self-efficacy.^29^ Some previous studies suggest thatfeedback improves self-efficacy and provides a feeling of proficiency. However, feedback may also be a source of anxiety**.**^7^ The experience of flow is another non-technical factor that appears to contribute to simulated and real-life laparoscopic learning.^30^*"When in flow an individual operates at full capacity".*^31^ Experiencing flow is to move or progress smoothly with unbroken continuity, with concentration and complete absorption in what you do. Flow depends on perceived action-capacities and opportunities. The balance between capacity and opportunity is, however, delicate. If a challenge begins to exceed your skills, you eventually become anxious. On the other hand, if your skills begin to exceed the challenges in the task at hand, you relax and run the risk of becoming bored.^31^ Thus, it is reasonable to assume that enhancing self-efficacy and flow in the operating room will potentially improve the surgical result and thereby patient safety. Additionally, assessments of these factors may be useful for evaluating feedback. In light of the controversies regarding feedback described above, the aim of this study was to explore the potential benefits of individualized feedback on the surgical result, self-efficacy and flow in a standardized setting involving simulated laparoscopy. In addition, we investigated students' perceptions of the learning process in a laparoscopic simulator with or without feedback during this learning processto collect information on how, when and how much feedback ought to be given.

## Methods

This was a mixed methods study investigating the value of individualized feedback on the simulated surgical experience and on self-efficacy and flow. Additionally we explored students' perception of learning in a simulator with and without instructor feedback.Data was collected from group interviews, questionnaires and from the simulator performance score.

### Setting

This study was conducted at the Center for Advanced Medical Simulation and Training (CAMST), Karolinska University Hospital, Stockholm, Sweden and was approved by the regional research ethics committee in Stockholm in September 2012.

### Participants

The study participants were medical students assigned to one of the OBGYN undergraduate courses, year 5, at Karolinska University Hospital, Stockholm, Sweden, during the fall of 2012. Participation in the study was voluntary. Laparoscopic simulator training was not part of the participants' academic curriculum, but was offered as an extra-curricular activity within the study. We chose medical students since they had no experience of laparoscopy and were novices to simulated laparoscopy. We obtained oral and written informed consent from each participant. Simulator results, interviews and questionnaires were de-identified upon transcription.

Sixteen medical students (eight females) were included in the study. The median age was 25 years (range 24-29). Ten of the students had one prior experience of simulator training, more than one year prior to participation in the present study. Gender, age, left-handedness and prior experience of computer games and simulator training were distributed equally in the two groups.

### Study design

The participants were randomized into two groups (simulator training with or without instructor feedback), balanced for gender. A mixed study method^32^ was used in order to evaluate learning with feedback by quantitative and qualitative methods. The surgical instruments pathways were measured in the simulated operation. We chose to measure this particular parameter since the complication risk is dependent on handling of the instruments, the length of the pathway of the instruments and the duration of surgery.^11^^, ^^33^^, ^^34^ Furthermore, a visual analogue scale and a validated questionnaire evaluated students' experience of flow and self-efficacy.^35^^,^^36^ Finally students' views on the learning process in the surgical simulator with and without feedback were captured by group interviews.

All participants had individual discussions with an instructor, (the same instructor for every participant), regarding a patient case with an extra-uterine pregnancy in preparation for the simulated surgery. The training session in the simulator started with the instructor performing a laparoscopic tubal occlusion, to demonstrate the simulator and its instruments. Each student then performed one laparoscopic tubal occlusion to get familiar with the simulator. The instructor continued the demonstration with a salpingectomy, the actual patient case. Each student subsequently performed three laparoscopic salpingectomies. The students randomized to the feedback group received feedback throughout the procedures. Feedback was given continuously, individualized by reinforcing and correcting each step. Feedback was also provided after each procedure, based on the parameter scores of each student. The control group performed the salpingectomies without feedback. Hence, they did not receive the feedback given by the simulator by the parameter scores or feedback given by the instructor. Feedback was evaluated by the parameter score in the simulator, by self-efficacy scores, by flow scores and furthermore by focus group interviews.

#### Quantitative data

##### Simulated laparoscopy

For laparoscopic gynecological simulation we used the LapSimGyn^®^ VR simulator (Surgical Science AB, Gothenburg, Sweden). The software runs on a Xeon-1.8 GHz processor using the Microsoft Windows^®^ XP (Microsoft Corporation, Redmond, WA, USA) operating system. The computer is equipped with 256 MB internal RAM, a NVIDIA Quadro2 EX graphics card (NVIDIA Corporation, Santa Clara, CA, USA), a 15-inch monitor and a virtual laparoscopic interface manufactured by Immersion Inc. (San Jose, CA, USA). Construct validity (to differentiate an expert from a novice)^37^and transfer of skills to the operating theatre have been established for LapSim^®^.^5^^,^^12^^, ^^38^ Simulator tasks performed were "tubal occlusion" and "salpingectomy". In the salpingectomy, the subject uses both hands to manipulate the instruments. When the task starts the system presents an ectopic pregnancy in the right fallopian tube. The students were instructed to use the bipolar grasper first, followed by the diathermy scissors. All students performed the surgery from the lateral/right side of the ectopic pregnancy, i.e. operating primarily with their right instrument, while the left instrument was practically static. The simulator calculates scores for *e.g. *instrument pathways, bleeding amount, tissue damage. The simulator parameter analyzed in the present study was right instrument path length (m).

### Self-efficacy and flow

Self-efficacy was self-assessed before and after the simulator training session using a 3-item questionnaire where each item was rated on a 7-grade Likert-type scale.^36^

*1."I am confident that I can handle the most difficult parts of the tasks during the simulator training/ future simulator training." *

*2."I will comprehend the meaning of the simulator tasks/ future simulator tasks." *

*3."I am confident I will succeed in future simulator tasks."*

The flow experience was self-rated immediately after the session. Two components of flow, Pleasure (4 items) and Concentration (4 items), were measured using 0 to 10 visual analogue scales. An overall flow experience index was calculated according to the results from Ghani and Despande,^35^ by the mean value of the Pleasure and Concentration components. The maximum score possible to achieve was 20.

### Analysis and statistics of quantitative data

Data analyses were carried out using JMP^®^ version 9.0.0 (SAS Institute Inc., Cary, NC, USA) for Mac OS X^®^ version 10.5.7 (Apple Inc., Cupertino, CA, USA). Mann-Whitney U was used to evaluate differences in self-efficacy, flow scores and surgical performance between two groups. Wilcoxon matched paired signed rank sum test was used to compare self-efficacy scores before and after training and surgical performance between sessions. A p-value <0.05 was considered statistically significant.

#### Qualitative data

##### Focus group interview

Focus group interviews^39^ were used to capture students' perceptions of simulator training, the learning process and more specifically on feedback. The interviews were carried out with each group individually. For logistical reasons the focus sessions were held one to seven weeks following training. This delay was equal between the two groups. Participants in the focus interviews were asked to describe their experience in general terms. We asked open questions in order to stimulate reflection. Examples of questions that were asked were; *"What did you experience?"*, *"What did you learn?"*, *"Did you get enough support?"*, *"Did the situation appear authentic?"* The interviews were recorded and later transcribed.

### Analysis of qualitative data

Interview transcripts were analyzed using an inductive, thematic approach.^40^ Transcripts were read several times in order to get to know the material. We went beyond the manifest content that was explicitly stated and interpreted the latent content.^41^ Data was coded inductively and subsequently grouped into themes.^22^

## Results

The primary findings in the study include a significantly shorter instrument pathway in the group receiving feedback. Based on the interviews, we conclude that the whole study group felt that feedback was essential. In contrast to the control group, the feedback group was more concentrated on the task compared to the control group and captured the whole procedure. The results are presented below with the quantitative simulator results first, followed by the results of the analyses of flow and self-efficacy and finally the results from the qualitative analyses, the students' perceptions subdivided by different themes.

### Findings from the quantitative analysis: Simulator performance

**Figure 1:**
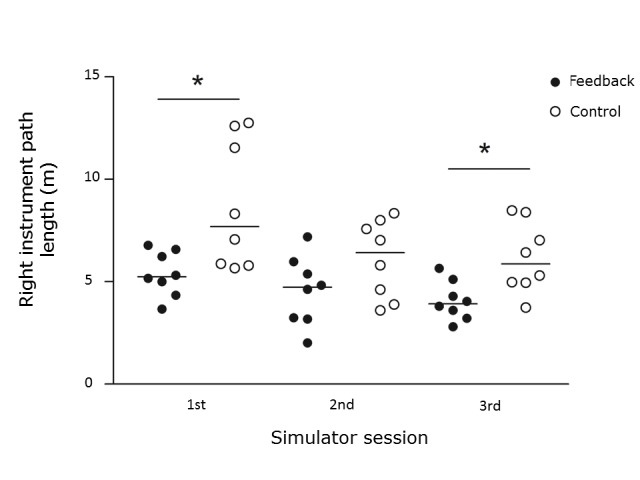
Simulator performance comparing groups. *p<0.05.

Each student performed three simulated laparoscopic salpingectomies.Right instrument path length was significantly shorter in the feedback group, in the first (median 5.2 m; IQR: 4.5-6.5) and last (median 3.9 m; IQR: 3.3-4.9) sessions as compared to the control group (median 7.7 m; IQR: 5.8-12.3) and (median 5.9 m; IQR: 5.0-8.1), p<0.05 (Figure 1). In addition, all students improved their performances significantly between the first and last session. The simulator performance was similar between male and female students.

### Flow and self-efficacy

**Figure 2:**
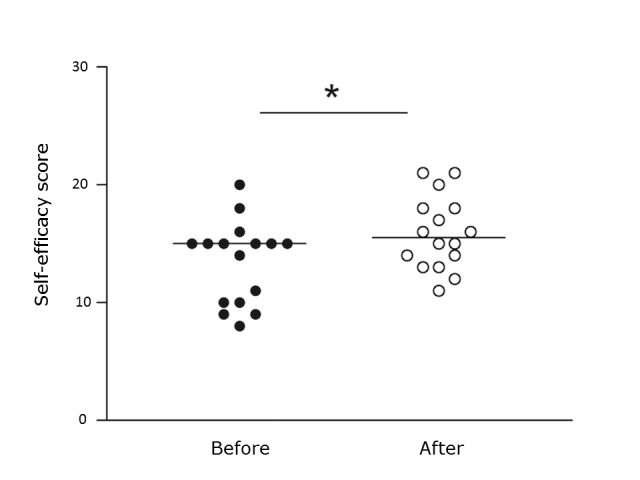
Self-efficacy score before and after simulator training. *p <0.05.

**Figure 3:**
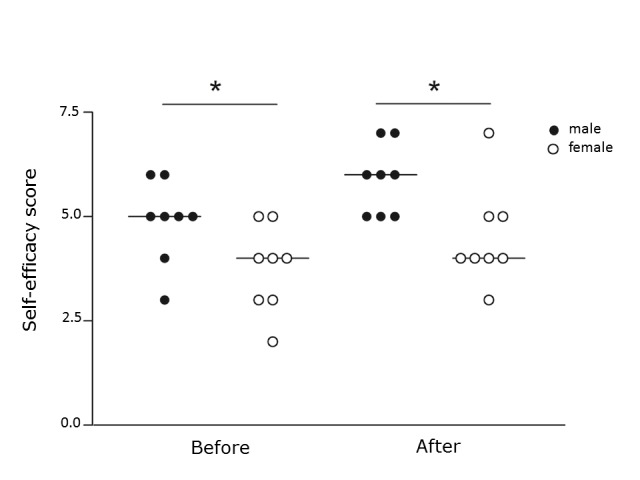
Self-efficacy score, item 3; "I am confident I will succeed in future simulator tasks", comparing males and females before and after training. *p <0.05.

Flow experience had a median score of 16.4 (range11.0-18.8) in the whole sample. No differences in flow experience or self-efficacy scores were observed between the feedback and control groups. Overall self-efficacy scores improved with training in both groups (Figure 2). Males scored significantly higher than females on self-efficacy item 3:"I am confident I will succeed in future simulator tasks", both before and after training (Figure 3).

### Findings from the qualitative analysis: Students' perceptions of the simulator training

Five to six students participated in each of the focus groups. Both male and female students were represented at each session. Three themes were identified: feedback, learning and experience/simulator training (Table 1).  The themes are described in more detail below.

**Table 1:** Focus interview results among medical students, Karolinska University Hospital, Stockholm, Sweden 2012

Themes	Codes	
	Feedback group	Control group
Feedback	Result improving every time; Supportive, Passive; Pressure to do well	Lack of feedback; Informative; Illustrative Lack of support
Learning	Understanding the procedure	Instrument handling
Experience/Simulator	Concentrated; Want to succeed and be acknowledged; Authentic Pressure to save the patient	Fun and exciting; Want to be seenGet support; Authentic Pressure to stop the bleeding

#### Feedback

This theme focused on the students' perceptions of feedback. The control group felt they lacked support and feedback. *"I definitely lacked support" *(Male 1, control group). *"You need an observer *(Female 1, Male 2, control group)*".*

The feedback group appreciated advice on how to think and how to do. They felt that each session in the simulator went smoother thanks to the feedback. *"I received feedback when I performed well and when and how I needed to improve my performance. Each subsequent session went smoother" *(Female 1, feedback group). In addition to from spontaneous feedback from the instructor, they also received feedback by asking questions. Some students expressed that they became passive and awaited initiative or acknowledgement from the instructor. Others expressed that the instructor was too passive and did not provide enough feedback. These students also asked for a more structured feedback.

#### Learning

What students learned from the simulator training constituted the second theme. The control group expressed that they appreciated an opportunity to handle the laparoscopic instruments, *"I got a feeling for how to handle the instruments" *(Female 1, Male 3, control group). They appreciated the procedure illustrations beforehand. They expressed that learning would have been enhanced if simulator training had been part of the mandatory curriculum and the exam.

The feedback group expressed that they better understood the surgical procedure. *"I came to understand the full procedure" *(Male 1, feedback group). They felt that the training fitted well within the context of the medical curriculum.

#### Experience of simulator training

The third theme focused on the experience of the simulator training. The control group expressed that simulator training was fun and even exciting. They became engaged in the activity, although not as much as if the training session had been part of the mandatory course. One student expressed; *"I had to stop the bleeding!" *(Male 4, control group)and another* "It was exciting!" *(Male 1, control group)Compared to the control group, the feedback group seemed to be more concentrated, but also more anxious. Several students expressed that they felt *"a pressure to do well"* (Female 2, male 2, feedback group) and to *"do the right thing for the patient." *(Male 3, feedback group)

Both groups perceived the session in the simulator as authentic, particularly the feedback group. The connection to the patient case was important for both groups.

Both groups expressed high ambitions to succeed and also the importance of support and being seen. The students in the feedback group expressed a more focused attitude towards the training, were more anxious not to succeed and experienced the situation as more realistic compared to the control group.

## Discussion

We here report beneficial effects of feedback on the performance in simulated laparoscopy. Our findings suggest that simulator training enhances self-efficacy and flow experiences. Participants in the control group expressed that they had more fun, whereas the feedback group was more concentrated on the task. Both groups had high ambitions to succeed and also expressed the importance of getting support and being acknowledged.

### Simulator performance

The feedback group used a significantly shorter path (i.e. moved the instruments less) compared to the control group, indicating a positive effect of feedback. Both groups, however, improved, regardless of feedback, which is in line with previous studies^37^^,^^42^ indicating an independent training effect of the simulator. The performances of the individuals in the feedback group were also less varied compared to the individuals in the control (Figure 1). This indicates that feedback is especially important for those students/trainees experiencing difficulties, perhaps in part due to a lesser visuospatial ability.^43^ Previous studies suggest that such individuals benefit the most from simulator training.^30^ This finding underlines the importance of individualizing feedback.

### Self-efficacy

In the current study, self-efficacy scores were similar between the two groups. Interestingly, these scores were significantly higher for males than for females concerning the item *"I am confident I will succeed in future simulator tasks" *both before and after training. Males did, however, not perform better than females in the simulator. Gender appears to affect the learning process and therefore needs to be considered during the construction of a curriculum.^44^ Self-efficacy scores increased for the whole group following training in the simulator, supporting the notion that simulator training has a positive effect on self-efficacy, as previously reported.^30^

### Flow

Both groups in this study scored relatively high on overall flow, suggesting that they approved of the simulator and its authenticity, regardless of feedback. The feedback group stated a high level of concentration, whereas the independent group stated more enjoyment. According to Ghani and Deshpande,^35^ concentration and enjoyment are key characteristics of flow, which can explain why no differences in the overall flow experience were observed between the groups and the lack of correlation with simulator performance. Whereas feedback appeared to enhance concentration and focus, despite a higher anxiety level, the independent approach seemed to enhance enjoyment and creativity.

### Learning

The students in this study stated that the authenticity of the situation, i.e. face validity (the simulator's ability to resemblance the real tool/ situation), was important for the learning process. Introducing the simulator session in the context of a patient case seemed to be an important factor.

The feedback group stated that they experienced a more realistic situation as compared to the control group. The feedback group was focused on the patient and the whole procedure, whereas the control group focused on procedural details, one at a time.

### Feedback

The instructor provided guidance and feedback to the feedback group during the simulator procedure, depending on the individual needs of each student. The parameter scores in the simulator also provided support for feedback on the performance. The control group received neither. The aim was to use constructive individualized feedback that is feasible and that can easily be transferred to the operating room. In a few cases, help was provided to allow the students of the control group to move on with the procedure, however, no feedback was given concerning these students techniques. This approach was chosen to somewhat mimic traditional surgical training and also to contrast the structured feedback.

The control group stated that they suffered from lack of feedback. On the other hand, some of the students that received feedback felt that it made them more passive or, as stated in some cases, that the instructor was too passive. The students in the present study wished for predetermined structures for both the instructor and the training. This is in line with the conclusions drawn by Evans et al. who report that the learning objectives need to be clear.^45^

### Experience of simulator training

Students in the feedback group expressed more anxiety as compared to the control group. They felt more pressure to succeed. This can be explained by the "mentor effect". Paskins et al. reported an increased anxiety level among medical students, during training with a whole body simulator, under teacher supervision.^46^ The students in the feedback group expressed an understanding of the whole procedure, whereas the control group was more focused on details, like instrument handling. The students in the control group felt more freedom to explore the instruments and their own capacity as well as the surgical approach. This indicates that students can benefit from an independent approach to learning. However, you cannot ignore the fact that technical skills improved more in the group that received feedback in this study. A mixture of feedback and independence might be the golden choice as indicated by Strandygaard et al.^25^ Moreover, feedback and being supported were factors that all the students felt facilitated learning.

### Limitations of the study

A limitation of this study is the relatively small study sample. The sample consisted of medical students and not residents in OBGYN, which is the main target-group for training and learning in gynecological laparoscopy. The reason for choosing novices was the advantage of their inexperience of simulators and lack of prior experience of laparoscopy, thus providing a homogenous test group. Another limitation is the way feedback was given. We did not use a validated tool, like for example Non-technical Skills for surgeons.^47^ A recent Danish study evaluated this tool adjusted for Danish circumstances in relations to feedback style.^48^ They found the tool useful, albeit still in need of improvement, for mentorship to reach its full potential. In the present study, we wanted the students' demands to guide the feedback style and to objectify it by using the parameter score in the simulator. We encourage future studies to use a tool/ checklist, but still allow the feedback to be adapted to the individual student and the specific situation.

Another limitation of the present study is the fact that we investigate the effect of feedback during simulated and not real laparoscopy and the findings can therefore not be generalized to the clinical situation. The simulator, however, provided a standardized patient scenario and procedure, which is impossible to obtain in a clinical setting. Different approaches to feedback in the operating room therefore need to be explored among residents in OBGYN.

## Conclusions

This study suggests that feedback enhances simulated laparoscopic learning. Students appear to benefit from support if it is carefully matched to their different needs. Compared to previous work, this study adds value as it highlights and meets the individual needs of feedback and explores students' views on feedback while learning laparoscopy. The next step is to optimize the amount and form of feedback and to transfer standardized and individualized feedback from the simulated setting to the operating room.

### Acknowledgments

The authors thank Mr. Armand Sadeghi, Department for Biomedical Engineering and Center for Advanced Medical Simulation and Training, at Karolinska University Hospital, Stockholm, Sweden for excellent technical assistance. This study was supported by research grants from the Stockholm County Council (FoUU and ALF).

### Conflict of Interest

The authors declare that they have no conflict of interest.
